# Modeling Parkinson’s Disease: Not Only Rodents?

**DOI:** 10.3389/fnagi.2021.695718

**Published:** 2021-08-06

**Authors:** Maria Shadrina, Petr Slominsky

**Affiliations:** Laboratory of Molecular Genetics of Hereditary Diseases, Institute of Molecular Genetics of National Research Centre “Kurchatov Institute”, Moscow, Russia

**Keywords:** Parkinson’s disease, pathogenesis, *in vivo* models, toxic models, genetic models

## Abstract

Parkinson’s disease (PD) is a common chronic progressive multifactorial neurodegenerative disease. In most cases, PD develops as a sporadic idiopathic disease. However, in 10%–15% of all patients, Mendelian inheritance of the disease is observed in an autosomal dominant or autosomal recessive manner. To date, mutations in seven genes have been convincingly confirmed as causative in typical familial forms of PD, i.e., *SNCA, LRRK2, VPS35, PRKN, PINK1, GBA*, and *DJ-1*. Family and genome-wide association studies have also identified a number of candidate disease genes and a common genetic variability at 90 loci has been linked to risk for PD. The analysis of the biological function of both proven and candidate genes made it possible to conclude that mitochondrial dysfunction, lysosomal dysfunction, impaired exosomal transport, and immunological processes can play important roles in the development of the pathological process of PD. The mechanisms of initiation of the pathological process and its earliest stages remain unclear. The study of the early stages of the disease (before the first motor symptoms appear) is extremely complicated by the long preclinical period. In addition, at present, the possibility of performing complex biochemical and molecular biological studies familial forms of PD is limited. However, in this case, the analysis of the state of the central nervous system can only be assessed by indirect signs, such as the level of metabolites in the cerebrospinal fluid, peripheral blood, and other biological fluids. One of the potential solutions to this problem is the analysis of disease models, in which it is possible to conduct a detailed in-depth study of all aspects of the pathological process, starting from its earliest stages. Many modeling options are available currently. An analysis of studies published in the 2000s suggests that toxic models in rodents are used in the vast majority of cases. However, interesting and important data for understanding the pathogenesis of PD can be obtained from other *in vivo* models. Within the framework of this review, we will consider various models of PD that were created using various living organisms, from unicellular yeast (*Saccharomyces cerevisiae*) and invertebrate (*Nematode* and *Drosophila*) forms to various mammalian species.

## Introduction

Parkinson’s disease (PD) is a common chronic progressive multifactorial neurodegenerative disease that affects at least 1% of people over the age of 65 and at least 4% of people over the age of 80 (Elbaz et al., 2016). It is characterized by the classic tetrad of motor symptoms (bradykinesia, resting tremor, rigidity, postural instability). However, in recent years, the diagnostic criteria for the disease have been refined, and postural instability is not among the obligative criteria for PD (Postuma et al., 2015). Motor symptoms are associated with impaired dopamine synthesis in the pars compacta substantia nigra as a result of the death of dopaminergic neurons and a decrease in the level of this neurotransmitter in the striatum. At the same time, it is believed that the appearance of motor symptoms is observed only with a very significant decrease in dopamine levels—up to 70–80% of the norm. The appearance of the first motor symptoms is preceded by a number of non-motor features (Jankovic, 2008a,b). These include, first of all, constipation associated with impaired motility of the large intestine, rapid eye movement sleep behavior disorder (RBD), impaired sense of smell, anxiety disorders, and anemia (Savica et al., 2010). These non-motor symptoms appear 15–20 years before the onset of motor symptoms. Non-motor symptoms are nonspecific, which prevents them from being used for the diagnosis of PD.

A typical pathological sign for PD is Lewy bodies and Lewy neurites—intracellular inclusions, the main component of which is the alpha-synuclein protein. A detailed analysis of these inclusions showed that the first such inclusions are formed long before the onset of the motor phase of the disease—at stages 1–2 according to Braak’s classification (Braak et al., 1998, 2000, 2004). At these stages, these inclusions are detected in the central nervous system in the dorsal motor nucleus, nucleus ambiguous, solitary nucleus, dorsal raphe nucleus, gigantocellular nucleus, olfactory bulbs, as well as in some types of intestinal neurons (for example, in the Auerbach plexus). Only at the third stage (also non-motor) Lewy bodies and Lewy neurites are detected in the substantia nigra, and at stages 4–6 they are detected in other areas of the nigrostriatal system, the mesocortex and the somato-sensory cortex (Braak et al., 2004). The concept of ascending development of lesions of the nervous system and frequent impairment of intestinal motility in the preclinical stage of PD have drawn attention to the important role of the gastrointestinal tract as a site of occurrence of the first inclusions of alpha-synuclein (Edwards et al., 1992; Braak et al., 2006; Sánchez-Ferro et al., 2015; Ambrosini et al., 2019). The gut microbiome may play an important role in the formation of the first alpha-synuclein inclusion bodies (Yang et al., 2019; Elfil et al., 2020).

In most cases, PD develops as a sporadic idiopathic disease. However, in 10–15% of all patients, mendelian inheritance of the disease is observed in an autosomal dominant or autosomal recessive type. It should be noted that according to the clinical picture, the sporadic and hereditary forms of the disease are practically indistinguishable. The first mutations in the familial form of PD were identified in 1997 in the alpha-sinulein gene *SNCA* (Polymeropoulos et al., 1997). To date, mutations in seven genes have been convincingly confirmed as causative in typical familial forms of PD, i.e., *SNCA, LRRK2, VPS35, PRKN, PINK1, GBA*, and *DJ-1*. Family and genome-wide association studies have also identified a number of candidate genes and common genetic variability at 90 loci, which have been associated with the risk of development of PD (Bandres-Ciga et al., 2020). Confirmation of the pathogenetic role of these requires further research. However, analysis of the biological function of both proven and candidate genes made it possible to conclude that mitochondrial dysfunction (Billingsley et al., 2019), lysosomal dysfunction, and impaired exosomal transport play an important role in the development of the pathological process (Bandres-Ciga et al., 2019, 2020). Immunological processes can play an important role in the development of the disease—this is evidenced by the association with the development of the disease of polymorphic variants in the BST1 gene and the HLA gene cluster. There is a hypothesis that it is immunological factors that can determine the predominant death of dopaminergic neurons (Billingsley et al., 2018).

However, despite all the successes achieved in the study of the pathogenesis of PD, the question of the mechanisms of initiation of the pathological process and its earliest stages remains unresolved. Understanding these mechanisms is necessary for the development of methods for the early preclinical diagnosis of the disease, as well as for the search for new approaches to pathogenetic therapy (Lotankar et al., 2017; Tarakad and Jankovic, 2017). The study of the early stages of the disease (before the first motor symptoms appear) is extremely complicated by the long preclinical period and the features of the pathology associated with damage to various parts of the central nervous system. In addition, at present, the possibility of conducting complex biochemical and molecular biological studies in persons with a reliably established risk of developing PD is limited. In fact, such an analysis can only be carried out in carriers of pathogenetically significant mutations in the genes of familial forms of PD. However, in this case, the analysis of the state of the central nervous system can only be assessed by indirect signs, such as the level of metabolites in the cerebrospinal fluid, peripheral, blood and other biological fluids.One of the possible solutions to the problem is the analysis of disease models, in which it is possible to conduct a detailed in-depth study of all aspects of the pathological process, starting from its earliest stages. A lot of modeling options are offered (Dauer and Przedborski, 2003; Danek et al., 2017; Ghatak et al., 2018; Kin et al., 2019; Chia et al., 2020). An analysis of studies published in the 2,000 s suggests that toxic models in rodents are used in the vast majority of cases (Kin et al., 2019). However, interesting and important data for understanding the pathogenesis of PD can be obtained from other *in vivo* models. Within the framework of this review, we will consider various models of PD, created using various living organisms—from unicellular [yeast (*S. cerevisiae*) and invertebrates (*Nematode*, *Drosophila*) to various mammalian species].

## Yeast Models: What Could Be Simpler?

Modeling of various pathological diseases in yeast cells has been actively developing since the last third of the 20th century (Mortimer, 2000). This type of model is aimed at studying the biological functions of the proteins associated with the development of monogenic forms of hereditary diseases and is based on the use of two main strategies. The first strategy consists of the heterologous expression of the genes of monogenic diseases (both wild-type genes and their mutant variants) in yeast cells. The second strategy entails the analysis of the functional activity in yeast cells of orthologous genes of pathologically significant human genes (Tenreiro and Outeiro, 2010; Dunham and Fowler, 2013). A combination of different approaches is also possible, e.g., the combination of the analysis of the orthologous gene of one of the disease genes with the heterologous expression of another pathogenetically significant gene (Menezes et al., 2015). Both of these strategies have been widely used in the study of genes responsible for monogenic forms of PD, i.e., the heterologous expression has been widely used for the analysis of the genes encoding the proteins parkin, PINK1, LRRK2, and alpha-synuclein. Orthologous gene analysis has also been used to study the biological function of human genes, such as *DJ-1*, *VPS35*, *EIF4G1*, and *ATP13A2*.

Alpha-synuclein is one of the proteins associated with the development of PD that has been most actively studied using yeast models. Concomitantly, it was shown that heterologous expression of the *SNCA* gene in yeast cells (in which there is no homologous gene) leads to a cytotoxic effect. The severity of this effect is determined by the level of expression of the *SNCA* transgene and the characteristics of the recipient yeast strain (Outeiro and Lindquist, 2003; Willingham et al., 2003). This cytotoxic effect is associated with the formation of intracellular inclusions, which are amyloid-like structures consisting of fibrillar alpha-synuclein, or the formation of vesicular inclusions (Soper et al., 2008). The formation of inclusion bodies leads to the disruption of various cellular processes, mainly the interaction of the endoplasmic reticulum with the Golgi complex, mitochondrial biogenesis, the functioning of proteasomes, mitophagy, and autophagy (Chen et al., 2005a,b; Dunham and Fowler, 2013; Menezes et al., 2015).

The important role of molecular chaperones in the development of alpha-synuclein toxicity was demonstrated using yeast models. It was found that the activation of the expression of genes encoding heat-shock proteins reduces the toxicity of alpha-synuclein aggregates, while the deletion of genes encoding individual chaperones increases their toxicity (Flower et al., 2005; Liang et al., 2008; Gade et al., 2014). The role of heat-shock proteins in the degradation of fibrillar alpha-synuclein was confirmed *in vitro* (Gao et al., 2015). Altered expression of the *HSC70* gene and several other genes encoding molecular chaperones was shown in the substantia nigra in patients with PD (Mandel et al., 2005; Grünblatt et al., 2018).

Genetic screening technologies using yeast models have shown that a very large number of biological processes modulate the toxicity of alpha-synuclein inclusions. Thus, the role of mitochondrial and proteasome dysfunction in alpha-synuclein-mediated cytotoxicity was confirmed. More than 80 new genes have also been identified that contribute to this type of toxicity. These genes have been associated with vesicular transport, lipid metabolism, ergosterol synthesis, and response to stress by the mTOR signaling pathway (Liang et al., 2008; Yeger-Lotem et al., 2009; Büttner et al., 2013; Hogg and Prehn, 2013). This significantly expanded our understanding of the possible role of alpha-synuclein in cells. However, the question remains as to how certain interactions between alpha-synuclein and other proteins/metabolic pathways play a role in the development of PD. The interactions identified in yeast models should be confirmed in other models of the disease and in patients with PD. A striking example of such a transition is the role of proteins of the VPS family (VPS24, VPS28, and VPS60) in reducing the toxicity of alpha-synuclein by controlling the sorting of proteins in the Golgi complex during the formation of endosomes and prevacuoles, which was first revealed in a yeast model (Liang et al., 2008) and was later confirmed by the detection of mutations in the gene encoding the VPS35 protein (a homolog of the yeast VPS35 protein) in one of the familial monogenic forms of PD, PARK17 (Vilariño-Güell et al., 2011; Zimprich et al., 2011; Ando et al., 2012).

Another aim of using yeast models with the formation of alpha-synuclein aggregates is the search for compounds that reduce the aggregation of alpha-synuclein monomers and/or the toxicity of the formed aggregates. The first such work was carried out by Su et al. (2010), in which the screening of a library of 115,000 compounds revealed a group of chemically related compounds of the 1,2,3,4-tetrahydroquinolinone family that reduced the formation of alpha-synuclein aggregates in yeast cells, followed by the normalization of transport between the Golgi complex and the endoplasmic reticulum and mitochondrial function. It should be noted that these compounds were active in preventing the formation of alpha-synuclein fibrils in other models, both *in vitro* (nematode neurons and rat midbrain neurons) and *in vivo* (toxic rotenone model of PD in rodents). These works led to the identification of a large number of compounds with a different chemical nature that reduce the toxicity of alpha-synuclein; i.e., mannosylglycerate (Faria et al., 2013), Latrepirdine (Dimebon; dimebolin) (Steele et al., 2013), several cyclic peptides (Kritzer et al., 2009), cerium oxide nanoparticles (Ruotolo et al., 2020), flavonoids, (poly)phenols, plant catechins [for example, from the leaves of *Arbutus unedo L*. (strawberry tree) and (green tea; Griffioen et al., 2006; Williams et al., 2007; Macedo et al., 2018), and yeast red pigment (a polymer of 1-(5-phosphoribosyl)-5-aminoimidazole; Nevzglyadova et al., 2018)]. The methodology used in this type of research is rapidly developing, and the use of new screening systems for bioactive compounds in yeast (Brás et al., 2019) will allow the identification of new low-molecular-weight compounds—blockers of the formation of protein aggregates.

One of the main genes of the familial form of Parkinson’s disease is the *LRRK2* gene, which encodes a leucine-rich protein kinase, dardarin. Mutations in this gene lead to the development of an autosomal dominant form of the disease and are associated with the formation of toxic gain-of-function variants of LRRK2 protein, which, in particular, were confirmed by the expression of various truncated variants of LRRK2 protein in yeast cells (Xiong et al., 2010). On the other hand, the LRRK2 protein can influence mitochondrial biogenesis in yeast cells. It has been shown that an increased level of this protein leads to a decrease in the level of de novo mitochondrial biogenesis and, thereby, to a decrease in the number of mitochondria in aging yeast cells (Aufschnaiter et al., 2018). A change in the number of mitochondria is also observed during the expression of various mutant variants of LRRK2 protein, which leads to an increase in the sensitivity of cells to hydrogen peroxide in yeast cells (Pereira et al., 2014). Thus, modeling in yeast made it possible to reveal important aspects of the functioning of LRRK2 protein and to reveal the key functions of this kinase (Seegobin et al., 2020).

## Modeling in Invertebrates: Simple Or Not

### Fruit Fly (*Drosophila*)

The advantages of the fruit fly as a model for studying various biological processes, including pathological ones, are obvious (Shulman et al., 2003; Bilen and Bonini, 2005; Freeman, 2015; McGurk et al., 2015; Zirin et al., 2020): (1) its short life span (about 1 month at an optimal temperature) which allows the tracing of pathological processes throughout ontogenesis; (2) its rapid sexual maturation and the ability quickly to generate a large number of progeny, which speed up the experiments; (3) its relatively small and well-studied genome and the availability of a large panel of methods for manipulating the fruit fly genome; (4) a large collection of fruit fly lines with various mutations; and (5) the conservatism of metabolic pathways and a similar organization of the nervous system (the presence of various types of glial and neuronal cells and the presence of a blood-brain barrier).

However, the nervous system of *Drosophila melanogaster* is not a complete analog of the human nervous system. A striking example of such differences is the alpha-synuclein protein. The gene that encodes this protein is actively expressed in the human nervous system, while an ortholog of the *SNCA* gene has not been found in the *Drosophila* genome. However, even in this case, the formation of inclusion bodies similar to Lewy bodies, the selective death of dopaminergic neurons, and locomotor disorders are observed in neuronal cells in flies that are transgenic for the human *SNCA* gene (Feany and Bender, 2000; Auluck et al., 2002, 2010; Chen and Feany, 2005). These processes are observed both regarding the expression of the wild-type *SNCA* gene and the expression of mutant forms of the *SNCA* gene with the missense mutations (A53T and A30P) identified in patients with the familial PARK1 form of PD (Lee et al., 2002; Dalfó et al., 2004). The death of neuronal cells is observed even when the *SNCA* transgene is expressed only in glial cells. Transgene expression in both neurons and glial cells leads to more severe dopaminergic dysfunction (Olsen and Feany, 2019).

Subsequently, genetic modeling was carried out for other hereditary forms of PD caused by mutations in the *PRKN* (parkin), *PINK1*, *LRRK2*, *VPS35*, *GBA* (beta-glucocerebrosidase), and *DJ-1* genes. Thus, a null mutation in the ortholog of the *PRKN* gene in *Drosophila* led to the disruption of locomotor activity and structural disorders in dopaminergic neurons. However, unlike humans, this *Drosophila* model also exhibited male sterility and myofilament apoptosis, which are caused by mitochondrial dysfunction (Greene et al., 2003; Pesah et al., 2004).

Later, it was shown that this effect may be associated with the activation of the JNK signaling pathway upon knockout of the *PRKN* gene, leading to the death of dopaminergic neurons (Cha et al., 2005). Conversely, pronounced mitochondrial dysfunction is associated with a decrease in mitophagy; in turn, parkin plays an important role in the induction of mitophagy (Cackovic et al., 2018).

A similar phenotype with a combination of male sterility, apoptotic death of muscle cells, and impaired mitochondrial morphology was detected in *Drosophila* with knockout of the ortholog of another PD gene, *PINK1* (Clark et al., 2006; Park et al., 2006; Yang et al., 2006). At the same time, a double knockout of the genes of the pink1 and parkin proteins led to a phenotype that was characteristic of the deletion of each of these genes separately. Conversely, overexpression of the *PRKN* gene minimized the consequences of the knockout of the *PINK1* gene, which indicates the participation of these genes in the same signaling pathway. It was later shown that the pink1 kinase activates parkin by its phosphorylation and the phosphorylation of ubiquitin. Activated parkin suppresses the mitochondrial adapter protein Miro in *Drosophila*, thereby reducing the activity of transport processes in mitochondria (Imai, 2020). The impairment of pink1 function can be at least partially normalized by various natural compounds, e.g., ginseng proteins (Liu et al., 2020).

Knockout models were obtained in *Drosophila* for the ortholog of the *LRRK2* gene (Lee et al., 2007). The deletion of this orthologous gene led to a decrease in locomotor activity, a decrease in tyrosine hydroxylase activity, and degeneration of dopaminergic neurons. Concomitantly, mutations of this type do not reproduce the real autosomal dominant missense mutations in *LRRK2* associated with PD. In this context, using the GAL4/UAS system, *Drosophila* lines were obtained that expressed the wild-type human *LRRK2* gene or carried the G2019S mutation in neuronal cells. Such transgenic flies exhibited degeneration of dopaminergic neurons, locomotor disturbances, and a decrease in life span, and these effects are more pronounced in transgenic flies carrying the G2019S mutation (Liu et al., 2008). The effects of the *LRRK2* transgene are highly dependent on the fly lines used for its expression, the genetic constructs, and specific mutations in the *LRRK2* gene. In different experiments, the effect of overexpression of the normal or mutant *LRRK2* gene on the phenotype is quite different (Imai et al., 2008; Venderova et al., 2009).

Imai et al. (2008) reported an analysis of the effect of the interaction between genetic and environmental factors in the development of PD. It was shown that the rotenone toxin enhances the development of the neurodegenerative process in *Drosophila* carrying the *LRRK2* transgene. However, it should be noted that, in general, studies of modeling of the sporadic form of the disease using the rotenone and 1-methyl-4-phenyl-1,2,3,6-tetrahydropyridine (MPTP) toxins are not widespread. This may be attributed to the difficulties in estimating the real dose of the toxin obtained in the simulation. Nevertheless, it was shown that (as in rodents) all of the toxins mentioned above reproduced the main features of PD, i.e., a decrease in locomotor activity and selective death of dopaminergic neurons (Coulom and Birman, 2004; Abolaji et al., 2018). These disorders are caused by oxidative stress resulting from the action of toxins, which can be partially stopped by antioxidants such as melatonin and resveratrol. Thus, toxic models in the future can be used to search for new antiparkinsonian drugs, provided that the methods of toxin introduction are standardized (Liu et al., 2020).

### Nematode *C. elegans*

The roundworm nematode has recently been widely used to study various biological processes. Similar to *Drosophila*, it is characterized by a short life span, a quick change of generations with a large number of progeny, ease of cultivation, and a relatively small and well-studied genome that is highly homologous to the human genome, as 40%–50% of all protein-coding nematode genes have orthologs in the human genome, including the orthologs of several genes that cause monogenic forms of PD (*LRRK2/lrk-1*, *PINK1/pink-1*, *PRKN/pdr-1*, *DJ-1/djr-1.1/djr-1.2*, and *ATP13A2/catp-6*). However, similar to that observed in *Drosophila*, an ortholog of the SNCA gene was not found in the nematode genome. Moreover, the introduction of the human SNCA transgene into *C. elegans* allows the modeling of the development of synucleinopathy. The genetic engineering methods used for working with the nematode genome are well established. For example, in the nematode, RNA interference methods are well developed, allowing the quick generation of a knockdown model of any gene of interest. For this, the transgene encoding the corresponding siRNA is introduced into the genome of the bacterial cells on which the nematode grows. Other methods of RNA interference have also been developed, including those that allow the tissue-specific knockdown of a gene of interest in a specific type of cell, e.g., in dopaminergic neurons (Firnhaber and Hammarlund, 2013). Working with *C. elegans* is also facilitated by the presence of a large collection of mutant nematode lines (at the Caenorhabditis Genetics Center^1^, where more than 20,000 strains have been deposited, including genetically modified strains and those expressing the fluorescent proteins GFP and YFP) and the ability quickly to obtain homozygous specimens for mutations in several genes of nematode lines because of their hermaphroditism (Dexter et al., 2012; Cooper and Van Raamsdonk, 2018).

The very high stability of the cellular structure of the nematode *C. elegans* is extremely important. Each hermaphroditic adult consists of exactly 959 somatic cells, 302 of which are nerve cells, which have been characterized in detail regarding their function and synaptic contacts using dopamine, serotonin, glutamate, and acetylcholine as neurotransmitters, as well as GABA.

The nematode is optically highly transparent, which allows the use of fluorescent proteins as trackers for individual genes, cells, or groups of cells, for the *in vivo* monitoring of their activity. Methods for the analysis of dopaminergic neuron dysfunctions in nematodes based on the analysis of worm mobility after starvation of varying duration have been developed and are termed “basal slowing response” and “enhanced slowing response.” Several other behavioral phenotypes have been proposed for analyzing the behavior of nematodes in the context of PD modeling, such as chemotaxis, area-restricted search behavior, swim-to-crawl transition, Dauer-dependent behavior, mechanosensory responses, fecundity, and rate of defecation (Maulik et al., 2017). In recent years, highly accurate methods have been developed for the analysis of motor activity and other aspects of nematode behavior based on the use of microfluidic technologies (Calahorro and Ruiz-Rubio, 2011; Wolozin et al., 2011; Dexter et al., 2012; Youssef et al., 2019).

The processes associated with the expression of the human *SNCA* transgene have been most actively studied (as in the case of *Drosophila*) in the nematode model. Several models overexpressing this wild-type gene (including those in which the gene is fused with the genes of the fluorescent proteins GFP or YFP) and gene variants with pathogenetically significant missense mutations under the control of different promoters specific for dopaminergic neurons and muscle cells have been reported.

It has been shown that some transgenic constructs in nematodes cause a selective decrease in the activity and death of dopaminergic neurons with the formation of inclusions of fibrillar alpha-synuclein, similar to Lewy bodies, in muscle cells. With age, these manifestations intensify (which is characteristic of PD) as the imbalance of protein homeostasis increases as the result of vesicular dysfunction, autophagy, and lipid metabolism disorders (Martinez et al., 2017; Ma et al., 2018; Gaeta et al., 2019). The severity of dopaminergic dysfunction depends on the number of copies of the introduced transgene, the features of its structure (wild-type *SNCA* transgene or missense mutations that cause PD), and the promoters used (their tissue specificity and potency). Several *C. elegans* genes have also been described that affect the cytotoxicity of transgenic synuclein and are associated with endocytosis (Lakso et al., 2003; Kuwahara et al., 2006; Gaeta et al., 2019). *VPS41*, which is expressed at a high level in dopaminergic neurons, is among the genes that were identified by siRNA screening. This gene encodes a protein involved in transport between the Golgi complex and lysosomes. As previously shown, its overexpression protects SH-SY5Y neuronal cells from the toxic effects of 6-hydroxydopamine and rotenone (Ruan et al., 2010). This indicates the possibility of using the *C. elegans* model not only for the analysis of known causative genes for PD but also for the search for new candidate genes for the disease.

An ortholog of gene *LRRK2* gene, *Lrk-1*, was found in the *C. elegans* genome. This gene is actively expressed in nematode nerve cells. Knockout of this gene does not lead to the death of dopaminergic neurons and pronounced motor impairments. On the contrary, a decrease in the level of dopamine with pronounced motor abnormalities is observed in transgenic animals carrying the human *LRRK2* gene with pathogenetically significant missense mutations. In this case, the death of dopaminergic neurons may be associated with a violation of both the kinase and GTPase activity of the LRRK2 protein (Seegobin et al., 2020). On the other hand, expression of the wild-type human *LRRK2* gene increases the resistance of the nematode to the toxic effects of rotenone and paraquat. These data indicate the importance of this kinase for the normal functioning of the organism (Langston et al., 2016). It would be extremely important to further study the role of LRRK2 protein in the *C. elegans* model using other pathogenic mutations.

In addition to genetic ones, toxic models of PD are widely generated using *C. elegans*. A large number of toxins have been identified that cause degeneration of dopaminergic neurons. The most actively studied toxins are 6-hydroxydopamine, MPTP, paraquat, and rotenone; however, several other compounds are toxic to dopaminergic neurons (for example, manganese ion and several pesticides; Jadiya and Nazir, 2012). In some cases, genetically modified nematode lines are required for the determination of toxic effects. Thus, 6-hydroxydopamine is active only in nematode lines expressing the dopamine transporter gene, which is necessary for the entry of the toxin into dopaminergic neurons (Nass et al., 2002). All of these toxins cause a decrease in the level of ATP in cells as a result of the development of mitochondrial dysfunction; in turn, damage to mitochondrial DNA replication can play an important role in the development of this condition (Zhou et al., 2013). In addition, toxins activate apoptotic processes and disrupt the folding of antioxidant proteins (Lehtonen et al., 2016; Offenburger et al., 2018).

Furthermore, *C. elegans* can be used to search for new toxins that cause degeneration of dopaminergic neurons. An analysis performed using *C. elegans* revealed the toxic effect of the heterocyclic amine beta-carboline, which is found in fried meat, roasted coffee, and tobacco (Sammi et al., 2018).

Several interesting results have been gathered from the study of the interaction between external factors and the orthologs of genes of monogenic forms of PD. This type of Gene × Environment (G×E) analysis not only affords a better understanding of the mechanisms of toxicity of various compounds but also allows the identification of natural and synthetic neuroprotectors that block, at least partially, the negative effects of the genetic damage associated with PD (Martinez et al., 2017; Garcia-Moreno et al., 2019).

The nematode can also be used to confirm the biological activity of small macromolecules identified by screening using other biological systems, such as yeast cells. For example, the analysis of yeast cells expressing transgenic alpha-synuclein led to the identification of *N*-aryl benzimidazole (NAB), which blocks the toxicity of alpha-synuclein through the activation of E3 ubiquitin ligase Rsp/Nedd4. The same activity was found for NAB in a nematode model overexpressing mutant alpha-synuclein. Moreover, NAB normalizes vesicular transport both in yeast and the nematode (Tardiff et al., 2013).

*C. elegans* modeling can be used to study the role of the microbiome in the development of PD. It was shown that metabolites of the soil bacterium *S. venezuelae* isolated from the culture medium cause several pathological disorders in the nematode that are characteristic of sporadic PD. In particular, mitochondrial dysfunction, impaired functioning of the proteasome system, and mitophagy processes were identified. Concomitantly, in the course of the development of the pathological process, there was a transition from a violation of individual metabolic processes to complex generalized disorders (such as mitophagy). Metabolites of other types of bacteria, including those that are characteristic of the human microbiome, can have a similar effect. Consequently, the nematode represents a convenient object for the search and detailed characterization of such metabolites (Caldwell et al., 2018).

## Step Forward: Vertebrates, from Simple to Complex

### Fish Models: Zebrafish and Medaka

Zebrafish (*Danio rerio*) and medaka (*Oryzias latipes*) are two fish species that are characterized by small size, rapid attainment of sexual maturity, relatively simple maintenance and reproduction, and the possibility of detailed analysis of motor behavior disorders (Orger and de Polavieja, 2017; Vaz et al., 2018). It should be noted that an overwhelming amount of work has been carried out using the zebrafish model, while practically only one laboratory works with medaka (Matsui et al., 2012, 2014; Matsui and Takahashi, 2018).

As vertebrates, fish are much closer to humans regarding the genomic organization and physiological characteristics than nematodes and *Drosophila*. The brain of these fish species consists of three sections (forebrain, midbrain, and hindbrain) and is separated from the remainder of the body by the blood-brain barrier. In the brains of these fishes, dopaminergic neurons were found in the posterior tuberculum, which can be considered as an analog of the substantia nigra from mammals. Genetically, zebrafish and medaka are closer to humans than are fruit flies and nematodes; for example, 70% of human genes have orthologs in the zebrafish genome, including orthologs of genes that cause the familial forms of PD (*LRRK2*, *PRKN*, *DJ1*, and *PINK1*). However, similar to *Drosophila* and nematodes, the zebrafish genome does not possess an ortholog of the alpha-synuclein gene, although it carries orthologs of other synuclein genes: those encoding β-, γ1-, and γ2-synuclein (Howe D. G. et al., 2013). Collections of *D. rerio* lines with various mutations have been created, which facilitate their use for research purposes; moreover, a database on this fish species is available^2^ (Howe K. et al., 2013). Methods for generating various mutations in the *D. rerio* genome have been developed. In addition, the technology based on morpholino antisense oligonucleotides is widely used because it allows the simple and quick generation of a knockout model of any gene and the tracing of the effect of this knockout on the development of an organism, starting at the one-cell stage.

For example, knockout of the β- and γ1-synuclein genes in zebrafish leads to motor impairment against the background of impaired development of dopaminergic neurons and a decrease in dopamine levels (Milanese et al., 2012). A similar phenotype is observed upon overexpression of γ1-synuclein. However, the most pronounced disturbances in the dopaminergic system are observed after overexpression of the human *SNCA* transgene in zebrafish (Prabhudesai et al., 2012), and these disorders are associated with dysfunction of the ubiquitin proteasome system. Thus, the *D. rerio* model can be used to analyze the complex relationships between the different forms of synucleins during the process of dopaminergic neuron death. In addition, a *D. rerio* model was used to analyze the molecular mechanisms of neurodegeneration associated with other genes responsible for monogenic forms of PD (such as *LRRK2*, *PINK1*, *ATP13A2*, and *PRKN*; Matsui et al., 2013a,b) and to search for genes associated with neuroprotection (Pinho et al., 2016; Hu et al., 2017; Feng et al., 2019). However, the results obtained in different studies may not coincide. Conflicting data were obtained using morpholine knockout and knockdown of the orthologous *LRRK2* gene. Depending on the dose of morpholine and the target domain in the LRRK2 protein molecule, both complete embryonic lethality and partial dopaminergic neuron dysfunction with the formation of inclusions of beta-synuclein in different types of neurons could be observed (Prabhudesai et al., 2016). Transgenic *D. rerio* carrying the wild-type human *LRRK2* gene exhibit increased locomotor activity, while the G2019S mutation disrupted the functioning of the ubiquitin-proteasome system in transgenic *D. rerio* (Seegobin et al., 2020). However, as in the case of the nematode, it is necessary to obtain and analyze a larger number of transgenic lines of zebrafish with various pathogenetically significant mutations.

In addition to genetic modifications, zebrafish are susceptible to various toxins that cause the selective death of dopaminergic neurons, such as the well-known MPTP, 6-hydroxydopamine, and rotenone (Sarath Babu et al., 2016; Wang et al., 2017; Zhang et al., 2017; Zeng et al., 2018). The effects of toxins vary depending on the scheme of drug administration (intraperitoneal administration or injecting a toxin into the water) and the stage of ontogenesis. This must be considered when modeling a pathological process and comparing the results obtained in different experiments.

The zebrafish model can be used to assess the potential neurotoxicity of normal metabolites, new pesticides, and other newly synthesized chemical compounds (Li et al., 2014; Lulla et al., 2016; Ren et al., 2016) as well as for the rapid screening of compounds with suspected neuroprotective activity. For example, the neurotoxicity of dopaminaldehyde (DOPAL), which is one of the metabolites of dopamine and L-DOPA, has been shown. These data draw attention to the question of the role of endogenous metabolites in the induction of neurodegenerative processes and indicate the importance of the selection of adequate therapeutic concentrations of L-DOPA, to prevent the formation of excessive amounts of DOPAL (Stednitz et al., 2015).

Conversely, zebrafish models, both toxic and genetic, have been used to screen for compounds with neuroprotective activity (Feng et al., 2016, 2019; Cronin and Grealy, 2017; Li et al., 2018; Soman et al., 2019; Wang et al., 2019; Ünal et al., 2020). Several protocols have been proposed for the rapid screening of such compounds (Pitchai et al., 2019). In addition, fundamentally new potential mechanisms for the prevention of neurodegeneration in PD have been identified; for example, the blockage of the expression of the histone deacetylase (HDAC) HDAC1 and HDAC6 (Pinho et al., 2016).

### Modeling on Mammals: Minipigs and Others

As mentioned above, an overwhelming amount of work aimed at modeling PD has been carried out in mammals, especially using mice and rats. The role of these rodent species is especially prominent in the toxic modeling of the disease because more than 95% of all such experiments have been performed using these models. Moreover, regarding genetic modeling, rodents occupy an honorable first place, as at least 80% of all work has been carried out using mice and rats (Kin et al., 2019). Nevertheless, PD modeling work has also been performed using other types of mammals, such as minipigs, dogs, and various species of primates.

The first report of PD modeling in Göttingen minipigs was published in 1999 (Mikkelsen et al., 1999). Moreover, it was shown that, in these animals, MPTP causes a decrease in the level of dopamine and the death of dopaminergic neurons in the substantia nigra, which leads to impaired motor behavior. Several different protocols of toxin administration have been proposed, and a micropump-based chronic continuous MPTP administration method has been developed that provides stable disruption of dopamine metabolism and behavior when administered at 12–18 mg of the drug per day for 11 weeks (Nielsen et al., 2016).

It is necessary to emphasize the high similarity of the brain structure between humans and minipigs, as well as the large size of the substantia nigra and striatum in minipigs. This facilitates the comprehensive molecular biological and biochemical analyses that are necessary for PD modeling (Wakeman et al., 2006; Nielsen et al., 2009). Their large brain size also allows the effective use of positron emission tomography analysis of brain structures, to monitor the development of neurodegeneration and changes in the nervous system, to correct neurodegenerative changes (e.g., the transplantation of embryonic mesencephalic neurons into animals with MPTP-induced parkinsonism; Danielsen et al., 2000). Large brain size also increases the ability to perform stereotaxic operations, for example, to obtain a 6-hydroxydopamine model of PD (Christensen et al., 2018). It has been shown that unilateral injection of the toxin into the nigrostriatal pathway induces a characteristic rotational behavior in minipigs, which is induced by the administration of amphetamine or deep brain stimulation on the affected side. Other toxic models of PD in minipigs have also been proposed, including a model based on the introduction of the proteasome inhibitor lactacystin into the medial forebrain bundle (Lillethorup et al., 2018).

Genetic models of PD were also obtained using Guangxi Bama minipigs based on the introduction of a human *SNCA* transgene carrying pathogenetically significant missense mutations (Zhu et al., 2018) or heterozygous knockout of three PD genes simultaneously (*PINK1*/*DJ-1*/*PRKN*, Wang et al., 2016). However, in both cases, transgenic minipigs did not exhibit any of the motor disorders that are typical of PD at the age of 3 and 10 months. Moreover, no PD-specific histopathological changes were detected in the substantia nigra and striatum. These findings may be attributed to the slow development of the pathological process in transgenic animals.

An extremely important area of research using minipigs is the analysis of new therapeutic approaches for PD because the great similarity of the anatomy and physiology between minipigs and humans allows a more accurate assessment of the relevance of the proposed treatment methods (Sousa e Silva et al., 2011; Lin et al., 2013; Ramot et al., 2017; Kahana et al., 2020).

Dogs, in which mutations in genes responsible for monogenic forms of PD have been described, may represent a promising research direction in the field of PD modeling.

For example, a mutation in the *ATP13A2* (PARK9) gene has been described in the Tibetan terrier, which results in the formation of a truncated protein variant. In the homozygous form, this mutation leads to the development of adult-onset neuronal ceroid-lipofuscinosis, the phenotype of which partially overlaps with the PARK9 phenotype of PD (Farias et al., 2011). It would be extremely interesting to develop such a PD phenotype in terriers heterozygous for this mutation. Other mutations have been reported in dogs that cause dysfunction of the brain regions associated with Parkinson’s disease. For example, the development of multiple systemic atrophy in the Chinese crested dog and Kerry blue terrier, which affects the substantia nigra, is associated with the genomic region in which the *parkin* gene is located in dogs (Wöhlke et al., 2011).

However, most importantly, dogs may be used in future research to study the role of the microbiome and the interaction between the brain and the gastrointestinal tract in the development of neurodegenerative diseases and, in particular, PD (Ambrosini et al., 2019). Dogs allow the study of the brain—gastrointestinal tract axis both *in vitro* (organoid cultures of the intestine, various variants of model constructs based on microfluidic technologies) and *in vivo*, especially given the relatively similar nature of nutrition between humans and dogs.

Nonhuman primates allow disease modeling in species more closely related to humans. Despite the obvious drawback of the high cost of work using primates, interest in these models has grown in recent years, primarily because primates can be used to develop new treatments for PD associated with the use of deep brain stimulation and transplantation of neuronal cells into the substantia nigra and striatum, as well as for testing newly developed drugs in the final stages of preclinical research (Fiandaca and Bankiewicz, 2010; Sundberg et al., 2013; Faggiani and Benazzouz, 2017; Wianny and Vezoli, 2017; Morissette and Di Paolo, 2018; Pignataro et al., 2018; Wichmann et al., 2018; Deffains and Bergman, 2019; Rosenblad et al., 2019; Teil et al., 2020). Moreover, the similarity of the organization of the nervous systems of humans and primates is fundamentally important because it allows the assessment in greater detail of the involvement of different brain structures in the development of the pathological process of PD, to analyze the temporal dynamics of this disorder (Verhave et al., 2011; Fifel et al., 2014; Vezoli et al., 2014; Ko et al., 2018; Sgambato and Tremblay, 2018; Wang et al., 2018; Deffains and Bergman, 2019; McGregor and Nelson, 2019). Nonhuman primate models also permit the simulation of the early, premotor, stage of PD; for example, sleep disorders (Bezard and Fernagut, 2014).

Previously, the injection of the MPTP toxin into primates was widely used in disease modeling (Fifel et al., 2014; Johnston and Fox, 2015; Rosenblad et al., 2019), to simulate the acute and chronic development of the pathological process in various nonhuman primates. Moreover, the exact dosage of the toxin to be administered to each animal is extremely important, with the possibility of correcting the amount of toxin injected according to the comprehensive assessment of the behavior of animals during the modeling process (Seo et al., 2019).

In recent years, a new direction of the work on the modeling of PD in primates is being actively developed in association with the use of alpha-synuclein as a toxin. In this context, various options for the introduction of alpha-synuclein were used. Koprich et al. (2016) injected the protein into the substantia nigra of cynomolgus macaques using the adeno-associated virus 1/2 (AAV1/2) vector carrying the transgene encoding human alpha-synuclein with the A53T mutation, thereby causing a decrease in the level of dopamine in the striatum and the death of dopaminergic neurons. It was later shown that the death of dopaminergic neurons with the formation of inclusions similar to Lewy bodies in TH-positive neurons can be caused in the common marmoset via the introduction of synthetic fibrillar alpha-synuclein into the region of the caudate nucleus or putamen (Shimozawa et al., 2017). Furthermore, the spread of the injected alpha-synuclein was observed in the brain, where it behaved as a prion-like protein. This spread was also observed after the introduction of alpha-synuclein (both as a fibrillar protein and a transgene in the AAV vector) into the neural plexuses of the intestinal submucosa. Alpha-synuclein inclusions were detected in the dorsal motor nucleus and locus coeruleus but were not accompanied by any symptoms of PD (except for intestinal dysfunction associated with damage to submucosal neurons; Manfredsson et al., 2018). Apparently, the results of the administration of alpha-synuclein depend greatly on the form in which it is administered, i.e., as a monomeric protein (in phosphorylated or dephosphorylated form) or as preformed fibrils (Gómez-Benito et al., 2020). The formation of Lewy bodies and neurites and interneuronal transmission of the introduced aggregates are observed only in the latter case. This indicates the importance of the onset of fibrillogenesis in the development of PD and the need to develop therapies aimed at blocking the formation of fibrillar alpha-synuclein or the interneuronal transmission of preformed fibrils (Hijaz and Volpicelli-Daley, 2020; Teil et al., 2020). In addition, the results of the introduction of different variants of alpha-synuclein isolated from the brains of deceased patients with PD into nonhuman primates show a complex picture for the development of neuronal disorders involving various brain structures. However, ultimately, nonhuman primates develop dopaminergic neuron degeneration (Bourdenx et al., 2020).

## Discussion

At present, a very large arsenal of models has been developed to study various aspects of the etiopathogenesis of PD *in vivo* using various model organisms, from unicellular eukaryotes to nonhuman primates. In this context, it is extremely important to select the optimal biological model for solving a specific problem.

**Figure 1:**
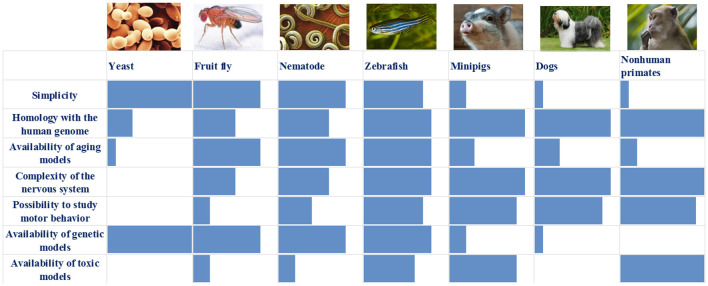
Comparison of the main characteristics of the described models: blue box—assessment of individual characteristics of the model (0–10 points).

We have summarized the main characteristics of all the models described above in Table 1 and Figure 1. It is obvious that each of the models has its own set of advantages and disadvantages that must be taken into account when choosing a model system for solving a specific problem. For example, the analysis of the individual stages of biological processes that are associated with the development of a disease (for example, autophagy or mitochondrial biogenesis) can be carried out using the simplest models, such as yeast, nematode, or *Drosophila*. In this case, new biological patterns can be revealed and key proteins that control specific biological processes can be identified. Such models are relatively simple from the point of view of the possibility of manipulating their genomes and creating complex genetic models, including the introduction into their genomes of mutant genes associated with the development of a pathological process in humans. Conversely, these simple models are genetically very distant from humans and their genomes may not contain the genes that are critical for the development of PD. A striking example is the genes encoding the proteins of the synuclein family, primarily the alpha-synuclein gene, which is absent in the genomes of all of the simpler model organisms. Because of this genetic remoteness, individual biological processes may proceed differently in model objects and in humans, and it is necessary to confirm the patterns identified in these objects using more complex models. It should also be noted that these models either do not have a nervous system (yeast) or have a nervous system that is very distantly related to the nervous system of primates and humans. Both *Drosophila* and nematodes have dopaminergic neurons, and their dysfunction can be actively studied in these models; however, the organization of neural networks with the participation of neurons of this type is fundamentally different both between these two models and between these models and primates.

**Table 1:** Brief characteristics, advantages, and disadvantages of the model objects.

	Yeast	Fruit fly	Nematode	Zebrafish	Minipigs	Dogs	Nonhuman primates
General characteristics of the model object							
Taxonomy	Unicellular fungi	Invertebrates, insects	Invertebrates, roundworms	Vertebrates, fish	Vertebrates, mammals	Vertebrates, mammals	Vertebrates, mammals
Life span	From several days to 2 weeks	About 2 month	15–25 days	Max. 2 years.	10–15 years	10–13 years	5–40 years for different species
The presence and complexity of the nervous system	Absent	The presence of various types of glial and neuronal cells (including dopaminergic), a blood–brain barrier, motor neurons, and interneurons	The presence of 302 nerve cells, which have been characterized in detail regarding their function and synaptic contacts using dopamine, serotonin, glutamate, acetylcholine, and GABA as neurotransmitters	The brain consists of three sections (forebrain, midbrain, and hindbrain) and is separated from the remainder of the body by the blood–brain barrier. Dopaminergic neurons were found in the posterior tuberculum, which can be considered as an analog of the substantia nigra in mammals.	The high similarity of the brain structure between humans and minipigs, as well as the large size of the substantia nigra and striatum in minipigs	The high similarity of the brain structure between humans and dogs, as well as the large size of the substantia nigra and striatum	The high similarity of the brain structure between humans and primates, as well as the large size of the substantia nigra and striatum
Possibility to study motor behavior	Absent	Climbing activity, pattern of movement, spontaneous locomotor activity	The analysis of worm mobility after starvation: “basal slowing response” and “enhanced slowing response”, Chemotaxis, area-restricted search behavior, swim-to-crawl transition, Dauer-dependent behavior, mechanosensory responses, fecundity, and rate of defecation.	Deficits in evoked swimming response (bradykinesia) Decrease in total distance moved and swimming velocity (bradykinesia) Increase in number and duration of freezing episodes (dyskinesia).	Analysis of motor behavior: walking test, Hurdle test, open field locomotor activity	Locomotor activity in open field, walking test.	Parkinson’s disease rating scale (PRDS) for primates.
Simplicity and cost	+ (The most simple and inexpensive)	+ +	+ +	+ + + +	+ + + + + +	+ + + + + +	+ + + + + + + + + + (The most difficult and expensive)
**Genetic models**							
Genome sequencing and annotation	YES	YES	YES	YES	YES	YES	YES
Similarity to the human genome	LOW	LOW	LOW	LIMITED	HIGH	HIGH	HIGH
Existence of genetic models	Exists	Exists	Exists	Exists	Exists, but not widespread	A natural mutation in *ATP13A2* gene	NO
The main genes studied, homologous to human	*SNCA, LRRK2, PRKN, PINK1, PARK7, VPS35, EIF4G1, ATP13A2*	*SNCA, LRRK2, PRKN, PINK1, PARK7, VPS35, GBA*	*SNCA, LRRK2, PINK1, PRKN, PARK7, ATP13A2*	*SNCA, LRRK2, PRKN, PINK1, ATP13A2*	*SNCA, PINK1/ PARK7/PRKN*	*ATP13A2*	NO
Methods	knockout, knockdown, and overexpression, transgenic (mutant) animals	knockout, knockdown, and overexpression, transgenic (mutant) animals	knockout, knockdown, and overexpression, transgenic (mutant) animals	knockout, knockdown, and overexpression, transgenic (mutant) animals; the technology based on morpholino antisense oligonucleotides.	knockout, transgenic (mutant) animals	A natural mutation	NO
Collection of mutant lines of animals	Yeast Insertional Mutant Collection	The presence of a large collection of fruit fly lines with various mutations.	The presence of a large collection of mutant nematode lines	The presence of collections of *D. rerio* lines with various mutations	Absent	Absent	Absent
Application area	Analysis of selected genes / proteins associated with the development of the disease. Primary screening for new drugs.	Analysis of selected genes / proteins associated with the development of the disease	Analysis of molecular mechanisms of neurodegeneration associated with genes of monogenic forms of Parkinson’s disease	Analysis of molecular mechanisms of neurodegeneration associated with genes of monogenic forms of Parkinson’s disease; search for genes associated with neuroprotection	Comprehensive molecular biological and biochemical analysis; positron emission tomography analysis of brain structures, to monitor the development of neurodegeneration and changes in the nervous system, to correct neurodegenerative changes	Comprehensive molecular biological and biochemical analysis; a study of possible microbiome involvement in neurodegeneration	NO
**Toxicity models**							
Existence	NO	Exists, but not widespread	Exists	Exists	Exists	NO	Exists
Toxins	NO	The rotenone and MPTP	6-hydroxydopamine, MPTP, paraquat, and rotenone	6-hydroxydopamine, MPTP, paraquat, and rotenone	6-hydroxydopamine, MPTP	NO	MPTP*SNCA*
Methods	NO	Addition of a toxin into the food	Addition of a toxin into the food	Intraperitoneal administration or addition of a toxin into the water	Unilateral injection of the toxin into the nigrostriatal pathway, a micropump-based chronic continuous MPTP administration	NO	Acute and chronic MPTP administration. *SNCA* as pure fibrillar protein or AV-based expression cassette
Application area	NO	Toxic models in the future can be used to search for new antiparkinsonian drugs	Search for new toxins that damage dopaminergic neurons; Analysis of biologically active substances; Study of microbiome	Screening for compounds with neuroprotective activity; Assessment of the potential neurotoxicity of normal metabolites, new pesticides, and other newly synthesized chemical compounds	Positron emission tomography analysis of brain structures for monitoring of the development of neurodegeneration, changes in the nervous system, and correction of neurodegenerative changes; the analysis of new therapeutic approaches for PD	NO	Development of new treatments for Parkinson’s disease associated with the use of deep brain stimulation, transplantation of neuronal cells into the substantia nigra and striatum, and for testing newly developed drugs in the last stages of preclinical studies
**Advantages and disadvantages**							
Advantages	Simple system. The ability to obtain various mutations in genes associated with the development of the disease. Possibility of studying protein-protein interactions, searching for new candidate disease genes. Rapid initial screening of new potential neuroprotective drugs	A relatively simple and well-established system. The presence of large collections of mutant lines. Possibility of using both genetic and toxic models. The presence of a well-studied nervous system. There are dopaminergic neurons. Short lifespan. Possibility to study the relationship between aging and neurodegeneration	Ability to use a wide range of genetic and toxic models. Well-characterized nervous system, presence of neurons of different energies. Possibility of analyzing motor behavior. Short lifespan and the ability to analyze neurodegenerative changes in animals of different ages. Ease of maintenance and reproduction, low cost.	Ability to use a wide range of genetic and toxic models. Sufficiently high homology with the human genome, there are orthologs for most of the genes associated with the development of PD. A detailed description of the nervous system, the presence of neurons of different energies. Possibility to analyze motor behavior.	The high similarity of metabolism in general and organization of the nervous system to human analogs. Possibility of visualization of substantia nigra during PET scanning. Possibility of using toxic and genetic models. Possibility of testing antiparkinsonian drugs and other therapies.	Natural mutations in the genes of familial forms of PD, which make it possible to study manifestations in ontogenesis.	Possibility to analyze the clinical phenotype as close as possible to the clinical phenotype of PD in humans. Development of protocols for new methods of therapy on animals as close to humans as possible.
Disadvantages	Unicellular organism. The nervous system is completely absent. There are no orthologs for a number of genes that are fundamentally important for PD, primarily, orthologs of the synuclein gene.	Lack of orthologs for a number of genes that are fundamentally important for PD, primarily orthologs of synuclein genes. A fundamentally different organization of the nervous system in comparison with vertebrates and humans.	Lack of orthologs for a number of genes important for the development of PD. The ortholog of the synuclein genes is absent. There are significant differences in the organization of the nervous system from higher vertebrates.	More difficult conditions for keeping in comparison with nematodes and fruit flies. Longer lifespan, which complicates the study of the effects of aging on neurodegeneration processes.	Very limited range of genetic models. Relative complexity and high cost of keeping the animals. Limited opportunities to study the role of aging in the development of PD.	A very limited range of natural mutations. Lack of genetic models with targeted modification of candidate disease genes and toxic models. Limited opportunities to study the role of aging in the development of Parkinson’s disease.	Toxic models only. Long duration of the experiment. Very high cost of modeling. Problems with using large samples. Limited opportunities to study the role of aging in the development of Parkinson’s disease.

This dictates the need for a transition to more complex models with an organization of the nervous system that is closer to that of humans and that affords the ability to analyze disorders of motor behavior, which is the main clinical sign of PD. However, models generated using rodents and *Danio rerio* have limited capabilities to manipulate the level of expression of specific genes of interest in individual organs and tissues at different stages of ontogenesis. Therefore, when working with these models, researchers actively turn toward toxic models of the disease. This allows going beyond the genes limited to the hereditary monogenic forms of PD and the identification of fundamentally new factors of etiopathogenesis. Moreover, working with these models is relatively cheap and fast because the full simulation cycle does not exceed several weeks or (in extreme cases) months. However, these animals allow very poor modeling of the early manifestations of PD, such as disturbances in fine motor processes, sleep, and dysfunction of the gastrointestinal tract. Their analysis, which is extremely important for the development of preclinical markers of the disease, is only possible using more complex model animals, from minipigs to primates. In this regard, the models based on minipigs can be underestimated—they are quite close to humans and allow complex experiments with various options for both genetic and toxic modeling of the disease. Of course, minipig models are inferior to primate models, but this is offset by the relatively simple maintenance and handling of minipigs at a much lower cost per animal. It is possible to use the minipig models successfully to analyze new therapeutic approaches for PD; for example, in the development of therapeutic methods based on the transplantation of neuronal cells.

It should be noted that the risk of developing PD increases dramatically in old age. Unfortunately, the age factor is currently poorly taken into account in disease modeling. In this regard, two model objects (*Drosophila* and *Nematode*) may be of the greatest interest. The short lifespan of these models makes it possible to assess the effect of aging on the development of neurodegenerative processes, which is extremely difficult to do in models with a long lifespan. In this case, it is always necessary to take into account the lifespan of individual lines of *Drosophila* and *Nematodes*, as well as the possible variability of this indicator within a separate line. Since the development of aging processes and the overall life expectancy are greatly influenced by environmental factors, it is necessary to strictly control the conditions of keeping model animals. This is especially important for animals with a short life cycle, where the influence of environmental factors is especially great (Cooper and Van Raamsdonk, 2018). Even in *D. rerio*, the study of the aging factor requires a very long time, although in this case, maintaining a sufficiently large fish population for more than one year does not seem to be an insoluble problem. In other model vertebrates, the estimation of the age factor turns out to be practically impossible, which once again emphasizes the importance of using a wide range of different model objects and different approaches to modeling. We would like to note again that there is currently no ideal model of PD that can reflect all aspects of this disease. These aspects include “the age of onset, the temporal speed of the disorder, nor the spectrum of problems and pathologies you see in the clinic in patients with PD,” as described by Roger A. Barker in a discussion with Anders Björklund (Barker and Björklund, 2020). Nevertheless, because it is impossible to study the processes occurring in nerve cells in humans *in vivo* at present, disease models are crucial in this field of research. We agree with Anders Björklund, who indicated that the PD models currently available allow a more detailed study of individual mechanisms of PD pathogenesis, and can also be used for the development of new drugs and approaches for the treatment of the disease (Barker and Björklund, 2020). From our point of view, in this case, the development of works with the wide use of two animal models from those considered above, namely, *Nematodes* and *D. rerio*, deserves maximum attention. In both cases, these are animals with a very well-studied nervous system, the organization of which at the cellular and molecular level is close to the mammalian nervous system, which makes it possible to transfer the results obtained to humans with a sufficient degree of confidence. In both cases, a variety of variants of genetic and toxic models are possible, which makes it possible to select the optimal parameters for modeling in accordance with the set of tasks. An important advantage of the nematode is its short life cycle, which makes it possible to take into account the role of the aging factor in the development of neurodegeneration in PD.

## Author Contributions

MS and PS reviewed the literature, discussed the concepts, and wrote the manuscript. All authors contributed to the article and approved the submitted version.

## Conflict of Interest

The authors declare that the research was conducted in the absence of any commercial or financial relationships that could be construed as a potential conflict of interest.

## Publisher’s Note

All claims expressed in this article are solely those of the authors and do not necessarily represent those of their affiliated organizations, or those of the publisher, the editors and the reviewers. Any product that may be evaluated in this article, or claim that may be made by its manufacturer, is not guaranteed or endorsed by the publisher.

## Footnotes

^1^ https://cgc.umn.edu/

^2^ http://zfin.org/
